# A single-cell imaging screen reveals multiple effects of secreted small molecules on bacteria

**DOI:** 10.1002/mbo3.176

**Published:** 2014-06-07

**Authors:** Jeanne Salje

**Affiliations:** 1 Harvard Medical School200 Longwood Avenue, Boston, Massachusetts, 02115

**Keywords:** Cell–cell communication, electron microscopy, *Pseudomonas aeruginosa*, secondary metabolites, small molecules

## Abstract

Bacteria cells exist in close proximity to other cells of both the same and different species. Bacteria secrete a large number of different chemical species, and the local concentrations of these compounds at the surfaces of nearby cells may reach very high levels. It is fascinating to imagine how individual cells might sense and respond to the complex mix of signals at their surface. However, it is difficult to measure exactly what the local environmental composition looks like, or what the effects of individual compounds on nearby cells are. Here, an electron microscopy imaging screen was designed that would detect morphological changes induced by secreted small molecules. This differs from conventional approaches by detecting structural changes in individual cells rather than gene expression or growth rate changes at the population level. For example, one of the changes detected here was an increase in outer membrane vesicle production, which does not necessarily correspond to a change in gene expression. This initial study focussed on *Pseudomonas aeruginosa*, *Escherichia coli,* and *Burkholderia dolosa*, and revealed an intriguing range of effects of secreted small molecules on cells both within and between species.

## Introduction

Microbes in their natural environments live in complex communities with large numbers of cells of both the same and different species. Soil bacteria can share a grain of soil with tens or hundreds of different microbes, while the gut microbiome is exposed to hundreds of different microbial species as well as the mammalian host cells. This complex environment is very different from standard laboratory conditions, and an important frontier in microbiology lies in understanding the nature of this environment and how it impacts the behavior of individual cells.

The presence of a mixed species environment can affect an individual cell type in a number of ways. The established presence of one or more species can prohibit the growth of a new species through competition or bacteriocidal activity, for example, in the case of the mammalian gut microbiome where a healthy gut flora can protect against microbial infection (Curtis and Sperandio 2011; Schuijt et al. 2013). Alternatively, two species can coexist in a niche and modulate one another's activity directly. For example, two of the major microbiome species bacteroides and firmicutes have been shown to significantly alter their gene expression profiles when grown together compared with when grown separately (Mahowald et al. 2009). Some coexisting species have evolved to support one another's growth, for example, in cross-feeding situations where one or both species provides essential nutrients for the other (Ramsey et al. 2011).

There are a number of mechanisms by which cells can modulate the common environment and thus impact the activity of neighboring cells. One indirect way is through competition for limited resources such as nutrients, whereby fast growing cells outcompete those growing more slowly, or force them to switch their nutrient profile (Mahowald et al. 2009). Cells can also directly target competing cells by the production of antibiotic small molecules, bacteriocins, or other antimicrobial toxins (Diggle 2010; Curtis and Sperandio 2011; Dufour and Rao 2011; Rutherford and Bassler 2012). Conjugation or other means of DNA transfer cause changes in the genetic library of nearby cells, or may simply provide a nutrient source. Finally, cells release a large number of small molecules, which can serve as signaling molecules, nutrient sources or toxins (Pesci et al. 1999; Waters and Bassler 2005; Camilli and Bassler 2006; Dufour and Rao 2011; Heeb et al. 2011). This last class of interactions was the focus of this study.

Bacteria secrete large numbers of small molecules, with some families such as *Pseudomonas* known to produce a large amount of a diverse set of secondary metabolites. The exact profile varies with growth stage as well as in response to specific signals, and in the case of *Pseudomonas aeruginosa* the GacS/GacA two-component regulatory system plays an important role in regulating the secretion profile (Kitten et al. 1998; Lapouge et al. 2008; Wei et al. 2013). Bacteria are known to change their secretion profiles in the presence of other species, and this likely occurs through a combination of direct and indirect signals. The complex and dynamic secretion profile of bacterial populations can be measured using high-performance liquid chromatography (HPLC) on cell supernatants (Winson et al. 1995; Pesci et al. 1999; Ortori et al. 2007; Gupta et al. 2011). Small molecules secreted by bacteria have been shown to play a number of roles. Among the best studied is quorum sensing, which is thought to sense population size and induce switches in cell growth and metabolism accordingly (Juhas et al. 2005; Ng and Bassler 2009; Frederix and Downie 2011). Other secreted molecules are involved in virulence, nutrient scavenging (such as siderophores that chelate iron) or are secreted as waste products.

*Pseudomonas aeruginosa* is a clinically important opportunistic pathogen that affects immunocompromised patients especially those suffering from cystic fibrosis (Mulcahy et al. 2013). It has a large genome, which probably reflects the ability to grow in a range of very different environments including, but not limited to soil and human lungs (Stover et al. 2000; Spencer et al. 2003). Small molecule secretion has been particularly well studied in *Pseudomonas* due to the large amount and range of molecules produced by this species.

This study began with the question: What is the environment seen by individual bacterial cells in their natural environment, and how does this affect the behavior of this individual cell? It is very difficult to measure the precise environment of an individual cell, especially when attached to a surface as is typical of many natural environments, as this will depend on the proximity and activity of nearby cells as well as diffusion rates of small molecules. This will be much slower in the thick mucus of the mammalian lung or gut, compared with standard laboratory media. It is also extremely difficult to determine the actual effects of compounds on neighboring cells. One major challenge lies in determining the appropriate readout for cell behavior, which could be gene expression, growth rate, or other changes.

This report presents a first step toward trying to dissect the effects of a complex mixed microbial environment on an individual cell. Compounds secreted into the supernatant of *P. aeruginosa* cells were purified. A method to grow bacteria cells directly onto an electron microscopy grid was developed, and a screen was designed that would test the effects of the addition of extracted small molecules on the morphology of cells grown in this way. A number of different phenotypes were identified in this way that would not be detected using classical high-throughput screening approaches.

## Experimental Procedures

### Bacteria strains and growth conditions

*P. aeruginosa* strain PA01 (gift from Steve Lory, Harvard Medical School), *Escherichia coli* strain MG1655, and *Burkholderia dolosa* strain C-10-0 (gift from Tami Lieberman, Harvard Medical School) were used in this study. This *B. dolosa* strain is a clinical isolate that is in the *Burkholderia cepacia* complex, and that lacks O-antigen. Bacteria were grown in LB broth at 37°C with 220 rpm shaking.

### Extraction and fractionation

*Pseudomonas aeruginosa* was grown in 1L LB at 37°C for at least 36 h, until the supernatant appeared green in color. The cultured cells were spun at 4000 rpm for 20 min to pellet the cells, and the supernatant was filtered using a vacuum filter with a 0.45 *μ*m pore size. The supernatant was extracted using ethyl acetate, and the extracted compounds were fractionated using a C18 sep-pak™ column. Acetonitrile and water were used as solvents, and fractions were collected at three acetonitrile concentrations: 15%, 50%, and 100%. Each of these fractions was further separated into 6–10 pools using a C18 reverse phase HPLC column. Fractions were dried down, weighed, and resuspended in dimethyl sulfoxide (DMSO) at a concentration of 5 mg/mL.

### Electron microscopy screen

*Escherichia coli, Pseudomonas aeruginosa,* or *Burkholderia dolosa* were grown to OD_600_ of 0.5–0.6. A humidified chamber was prepared by placing parafilm together with a water-soaked kimwipe inside a petri dish. A quantity of 20 *μ*L bacteria was placed on the parafilm, together with 1 *μ*L DMSO or fractionated compound. Compounds were added at a final concentration of 250 *μ*g/mL. A glow-discharged, carbon-coated electron microscopy grid was placed on top of the droplet of bacteria, and cells were allowed to grow for 4 h at room temperature (around 25°C). After incubation, samples were wicked from the back, then grids were washed quickly in two drops of water and stained with 1% uranyl formate. Samples were imaged on Tecnai (Eindhoven, The Netherlands) G^2^ Spirit BioTwin equipped with an AMT 2K CCD camera. Large numbers of images were acquired for each condition, and these were analyzed manually. For analysis of vesicle number, the number of vesicles in at least three images was counted for each condition, and converted to average number per *μ*m^2^.

## Results

### Development of an assay to test for morphological changes in cells induced by the presence of naturally secreted small molecules

An assay was designed that would detect changes in cell structure when grown in the presence of different naturally secreted small molecules. Electron microscopy was selected as a detection tool due to the high resolution of imaging. This is a relatively slow technique and therefore sample number was limited to around 60 samples per experiment.

First, a method was designed to grow cells directly on carbon-coated electron microscopy grids, as this would better mimic many environmental growth conditions. It was observed that when cells were grown in this way they exhibited certain characteristics such as higher numbers of piliated cells, consistent with a switch to surface-adhered lifestyle.

The supernatant of growing *P. aeruginosa* cells was fractionated. It is known that secretion profiles are growth stage-dependent and in all experiments shown here the supernatants of cells in stationary phase growth was used. Cells undergo a quorum sensing-regulated switch after which larger numbers of small molecules can be detected in the medium. This is accompanied by a change in color to dark green, due to synthesis of pyocyanin, and this was used as an indicator of growth stage (Jayaseelan et al. 2013). This color change occurred after around 36 h of growth, and at this stage much higher yields of compounds could be extracted compared with earlier stages of growth. The total supernatant (2–8 L) was extracted with ethyl acetate, and the extract was further fractionated using a C18 sep-pak™ column with acetonitrile and water as solvents. A total of three fractions was collected (15%, 50%, 100% acetonitrile in water). Each of these three fractions was subjected to reverse phase C18 HPLC purification and further subfractionated into 6–10 fractions. Spectra from the HPLC purification of extracts and LB-only controls are shown in Figure S1. The fraction step size was limited to the number of experiments that could be performed in each screen.

**Figure 1:**
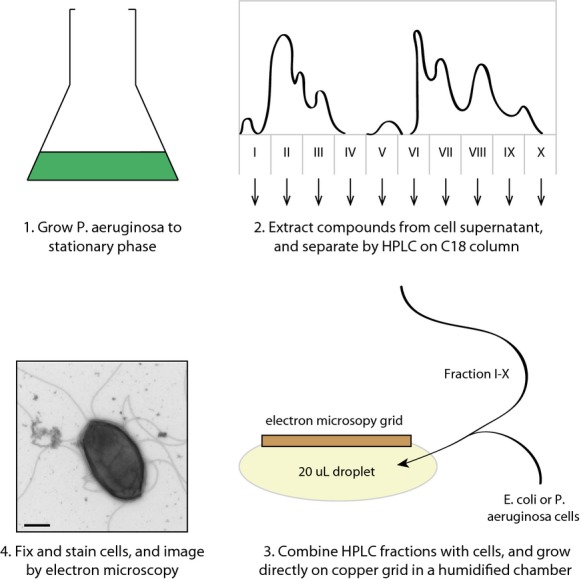
Figure showing experimental outline. (1) *Pseudomonas aeruginosa* was grown to stationary phase. (2) compounds were extracted from the supernatant and separated using reverse-phase chromatography. (3) Eluted fractions were added to growing *Escherichia coli, Burkholderia dolosa* or *P. aeruginosa* cells and grown on a electron microscope slide. Samples were fixed and (4) imaged by transmission electron microscopy.

The small molecule imaging screen was carried out in the following way (Fig.1). *P. aeruginosa, E. coli* or *B. dolosa* cells were grown directly on the grid inside a humidified chamber. To the cells was added nothing, DMSO, or one of the purified fractions from *P. aeruginosa* supernatant (resuspended in DMSO). Cells were grown for 4 h at room temperature and then fixed and stained for imaging. Cells treated with compounds were manually imaged and screened for morphological changes compared with untreated or DMSO-treated cells (Table1). Fractions shown to induce robust morphological effects were repeated and reimaged.

**Table 1:** Overview of responses by *Pseudomonas aeruginosa, Escherichia coli*, and *Burkholderia dolosa* to different purified fractions.

Fraction	Effect on *P. aeruginosa*			Effect on *E. coli*		Effect on *B. cenocepacia*	
	Outer membrane vesicles	Extracellular protein filaments	Budding volcanoes	Outer membrane vesicles	Ghost cells	Outer membrane vesicles	Ghost cells
Crude extract	**+**	**+**	**+**	**+**	**+**	**+**	**+**
Aqueous fraction							
15% Fraction							
50% Fraction							
100% Fraction	**+**	**+**	**+**	**+**		**+**	
15% VI				**+**		**+**	
50% FII		**+**					
50% FIV	**+**			**+**		**+**	
50% FV	**+**			**+**		**+**	
100% FI		**+**		**+**		**+**	
100% FII			**+**		**+**		**+**
100% FIV				**+**		**+**	
100% FV	**+**			**+**		**+**	

### Induction of outer membrane vesicle formation

**Figure 2:**
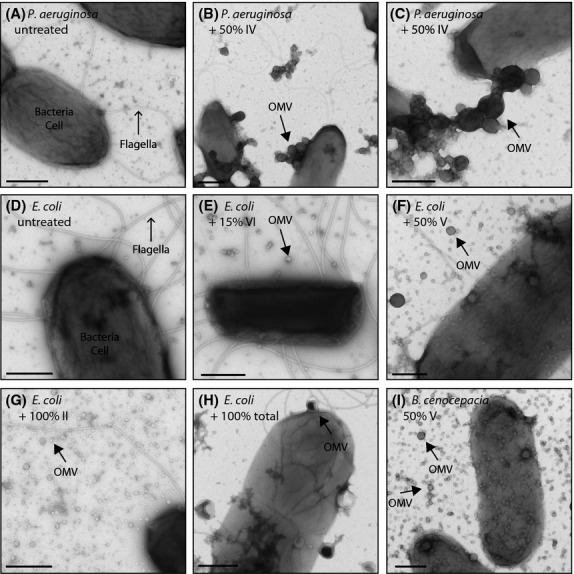
Electron microscopy images showing upregulation of OMV production in response to addition of certain purified compounds. The target cell and compound fraction is shown in each panel. Bacteria cell and flagella are indicated in the untreated panels for clarity. Block arrows indicate representative outer membrane vesicles. Scale bar = 500 nm.

**Figure 3:**
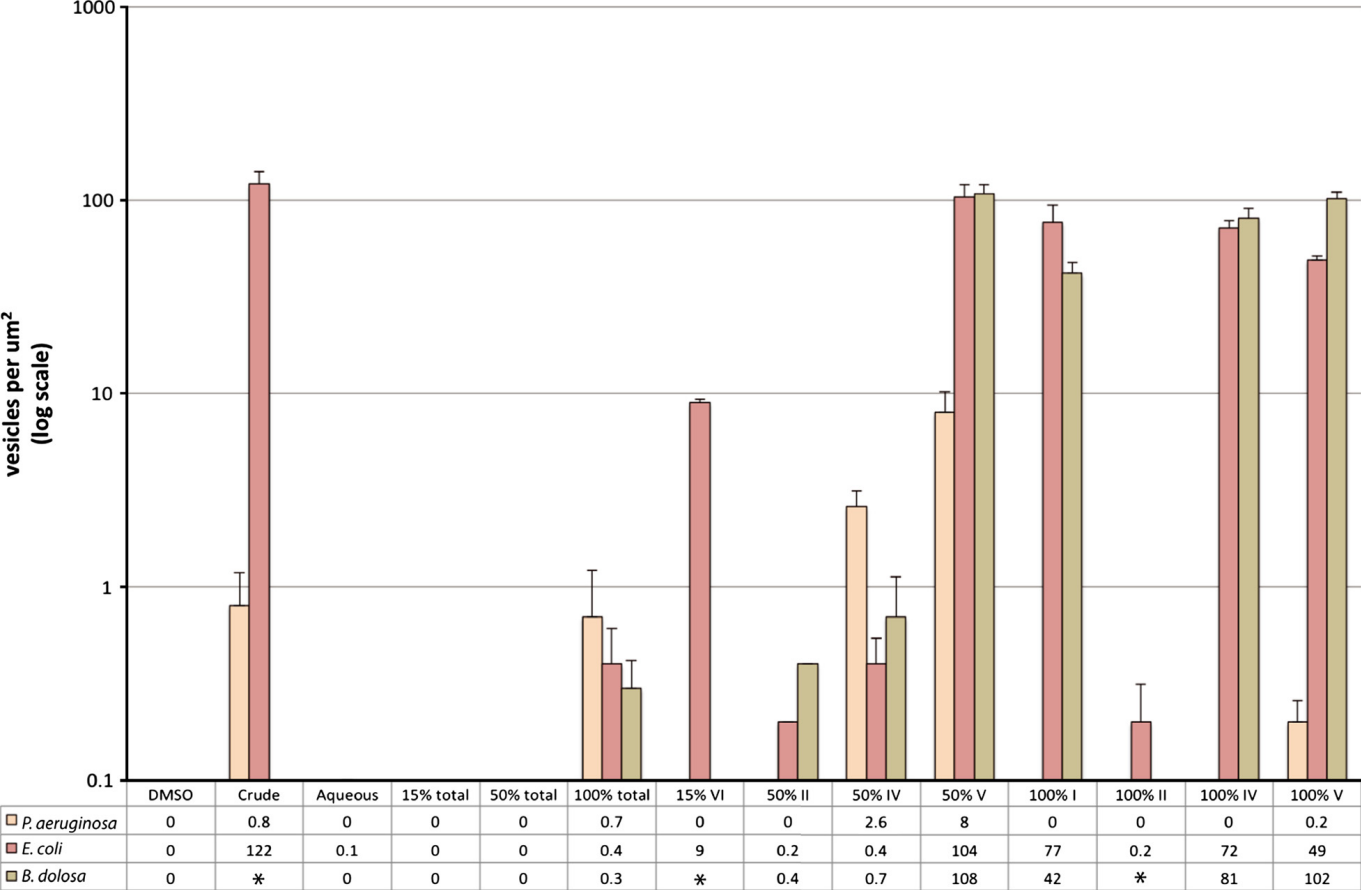
Quantification of outer membrane vesicle production. Graph showing the number of vesicles detected in electron microscopy images, shown as vesicles per *μ*m^2^. Average numbers (from at least three separate images) are shown below the graph, and error bars indicate the standard deviation. From left to right, bars represent vesicles produced by *Pseudomonas aeruginosa, Escherichia coli,* and *Burkholderia dolosa* in response to the fractions shown below. *indicates data not available.

A number of fractions contained compounds that caused upregulation of outer membrane vesicle (OMV) production (Figs.3 and Table1). The size, appearance and number of vesicles differed markedly between different fractions (Fig.2, 3). Interestingly, the OMV profile for each fraction differed between *P. aeruginosa* (self) and *E. coli* (non-self) both in terms of the fractions that induced OMV formation and in the morphology of OMVs imaged. For example, fraction 15% VI and fractions 100% I and IV induced OMVs in *E. coli* but not in *P. aeruginosa*. Equally, *E. coli* tended to produce a larger number of smaller vesicles compared with *P. aeruginosa*. This was compared with *B. dolosa,* which is a Gram-negative bacteria that coexists with *P. aeruginosa* in human lung infections (Jones et al. 2004; Bragonzi et al. 2012), and this showed a profile similar to that of *E. coli* (Table1, Fig.3).

In certain cases there were differences between the effects observed using crude fractions or finer fractions, for example, between the 50% total, and fraction 50% IV (Fig.3 and Table1). This may be due to different concentrations of individual components in the different mixes, or synergistic/antagonistic effects between compounds that become separated in finer fractions.

### Extracellular protein secretion

**Figure 4:**
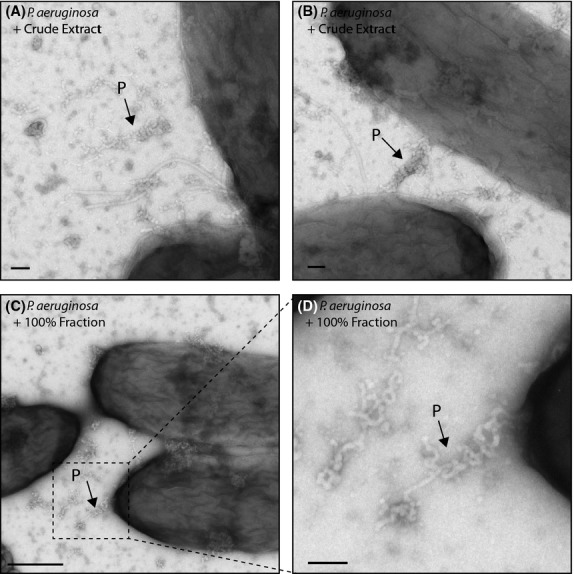
Electron microscopy images showing production of secreted protein in response to addition of certain purified compounds. Arrows highlight representative examples of protein (P). Scale bar = 100 nm (A, B), 500 nm (C) or 50 nm (D).

A number of fractions induced the formation of proteinaceous-looking elongated structures in *P. aeruginosa* (Fig.4). These were never observed in untreated or DMSO-treated negative controls, nor in any other cell types that were imaged. The structures typically appeared close to the edges of cells, and the cells did not appear damaged or exploded in any way. This suggests that this represents a structure that was secreted intact from the cell, perhaps through an upregulation of a secretion system. Attempts were made to identify the nature of this material through purification, but it was not possible to obtain sufficient quantities for mass spectrometry analysis. This may be a consequence of the difference between growing cells in a surface-adhered rather than planktonic state, since the latter was used when trying to purify secreted material.

### Membrane budding ‘volcanoes’

**Figure 5:**
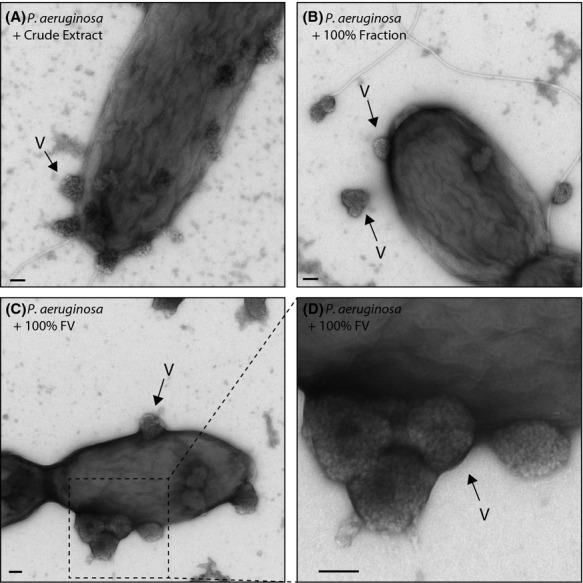
Electron microscopy images showing blebbing of the outer membrane of *P. aeruginosa,* into structures described here as volcanoes and indicated by arrows (V). These were formed in response to certain purified compounds, indicated on the panels. Scale bar = 100 nm.

One of the most unusual structures identified in this screen was the production of structures described here as ‘volcanoes’ which were observed budding off the sides of *P. aeruginosa* cells in a small number of fractions. These were only observed in *P. aeruginosa* cells, and never in untreated or DMSO-treated negative control samples. When present, these structures numbered 2–16 per cell and measured roughly 200 nm in diameter (Table S1). They were usually found tightly adhered to cells, but sometimes observed a small distance away (Fig.5). The cells did not appear otherwise damaged or exploded in any way. Similar to the secreted structures described above, attempts were made to purify these structures for analysis but they could not be produced in sufficient quantities.

### Cell ghost formation

**Figure 6:**
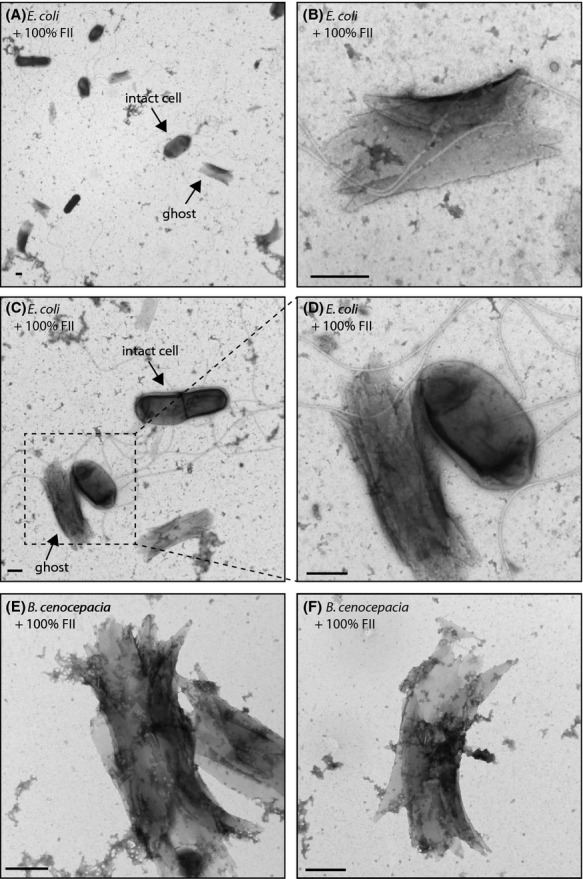
Electron microscopy images showing the formation of structures in *E. coli* and *B. dolosa* described here as ghosts. These formed in response to certain purified compounds which are noted on each panel. Scale bar = 500 nm.

A small number of fractions caused the formation of structures described here as ‘ghost’ cells, and these were observed in *E. coli* and *B. dolosa* but never in *P. aeruginosa* (Fig.6). These structures were the same size as intact cells, and appeared to be the empty shell of an exploded cell. The fractions that produced this phenotype did not destroy all the cells in a sample, but the effect on an individual cell appeared to be all-or-none. Within one sample there were a large number of intact cells and ghost cells, but no notable intermediate stage. One interesting feature of this apparent bacteriocidal effect is that it suggests a mechanism different from that of lysozyme treatment. Lysozyme digests the peptidoglycan in the cell wall, and lysozyme-treated cells in electron microscopy appear punctured all over and in various stages of lysis. The feature described here results in a neat membrane structure, possibly comprised of an outer membrane that has been hollowed out in the inside. Similar structures were never observed in any negative controls, but the same fractions gave very similar structures when added to growing *B. dolosa* cells suggesting a similar non-self-susceptibility (Fig.6). The effect of this fraction was measured at the population level by counting the number of colony-forming units in treated and untreated cells, and no significant effect on population size was detected (data not shown). While this may reflect slight differences in growth conditions between the different types of experiments, it may also highlight one of the advantages of this screen that is the ability to identify low frequency events that have little impact on the population level but significant impact on an individual cell.

## Discussion

Here, a screen was designed that would probe the effects of secreted small molecules on neighboring cells. Electron microscopy was used as a screening tool because it would detect physical changes in cell structure as well as events that occur at low frequency. The supernatant of *P. aeruginosa* was fractionated and tested for effects on *P. aeruginosa* itself as well as *E. coli* and *B. dolosa*. High concentrations (250 *μ*g/mL) were deliberately used to try to mimic what a cell might see when positioned in tight proximity to a neighbor. It was also reasoned that very high concentrations might cause an extreme response that would be easy to detect and would give information about more subtle effects that could occur at more physiological concentrations. The effect of *P. aeruginosa* extracts on itself was studied to try and isolate cell–cell interactions within a population. This was compared with the effect on *E. coli* as a generic Gram-negative bacterium with similar membrane structure, and also with *B. dolosa,* which is found together with *P. aeruginosa* in human lung infections (Jones et al. 2004; Bragonzi et al. 2012).

This screen has a number of limitations. First, it only detects those features that are physically adhered to the carbon surface of the microscopy grid, as well as those that are large enough to be detected by electron microscopy. Thus, any features smaller than ∼20 nm will be missed, such as small globular monomeric proteins. It also selectively detects those macromolecules that can be stained by the uranyl formate stain used here, and thus structures composed predominantly of lipid or carbohydrate could be missed. A second limitation is that effects may be missed due to the specific details of the compounds that are included in the screen. For example, the concentrations used may be too low or too high to elicit a response, different preparation methods may yield a different profile of compounds, and synergistic effects of compounds separated by fractionation may enhance or inhibit activity. Finally, the screen is subject to the limitations of low throughput of electron microscopy preparation and imaging.

In spite of these limitations this initial screen revealed some intriguing phenotypes. The four most robust and reproducible effects were presented here, and include an upregulation of OMV production, secretion of protein-like filaments outside the cell, production of budding ‘volcano’ structures outside the cell, and production of ‘ghost’ structures. A significant number of fractions induced an upregulation of OMV production in all three cell types. OMVs are common features of Gram-negative bacteria, and are known to play various roles in cell–cell communication as well as pathogenicity (Kadurugamuwa and Beveridge 1999; Mashburn and Whiteley 2005; Bauman and Kuehn 2006; Schertzer and Whiteley 2013). One of the major secreted compounds from *P. aeruginosa,* Pseudomonas Quinolone Signal (PQS) is known to play an important role in OMV formation (Diggle et al. 2006, 2007; Palmer et al. 2011). Preliminary analysis by mass spectrometry analysis of the compounds present in the fractions used here did indeed reveal the presence of various derivatives of PQS in a number of vesicle-inducing fractions. However, certain fractions did not contain detectable levels of PQS, and included significant amounts of other compounds including 4-hydroxy-2-alkylquinolines (HAQ). Furthermore, when the assay was repeated using equivalent concentrations of pure PQS it was found that the levels of vesicles detected by electron microscopy were much lower than when adding fractions we had purified. Taken together, this suggests that a number of other compounds are likely important in the synthesis and regulation of OMV formation by *Pseudomonas* both within the species and when targeting other species.

One interesting observation to emerge from an overview of these results is the different effects that the same purified fraction can have on different cell types. In the case of OMV production, the same fractions induced markedly different numbers and sizes of vesicles in *P. aeruginosa* compared with *E. coli* and *B. dolosa*. All three cell types are Gram-negative, rod-shaped bacteria of similar dimensions and with similar outer membrane properties. Indeed, the similarities between *P. aeruginosa* and *B. dolosa* are such that it was only recently that they were reclassified as different species. There are two possible mechanisms by which a small molecule can induce a change in the cell structure such as the formation of OMVs or ghost cell structures. First, the compound may bind directly to the outer membrane and induce structural deformation leading to membrane budding or lysis. This mechanism has been proposed for PQS (Schertzer and Whiteley 2012). The fact that the same fraction has different effects on cells with similar membrane properties suggests that the budding process may be affected by the presence of specific proteins or carbohydrates in the outer membrane. Second, the compound may induce intracellular signaling either through binding to a signaling receptor on the surface or through uptake and cytoplasmic signaling. It is likely that different compounds use different mechanisms for activation.

This screen presents a first attempt to isolate certain structural effects induced by naturally secreted small molecules. This revealed four robust phenotypes that are suitable for follow up investigation, which will include molecular identification of secreted structures, development of bioassays to test for the presence of these features in genetic mutant libraries, and identification of active compounds by finer fractionation of the *P. aeruginosa* supernatant. Presented here is a proof of principle that this approach can reveal new mechanisms of cell–cell interactions, and this approach can easily be expanded to include more compounds, finer fractions, different growth stages, and combinations of different cell types.

Bacteria exist in a mixed environment with other species, and it is evident that cell–cell interactions govern growth and metabolism of cells at a population level. It is intriguing to imagine how such interactions are affected at the molecular and cellular level, but it is very difficult to dissect how cells impact one another's activity within a complex population. Here, I propose one approach to try to unravel the exciting question of how cells communicate on the level of individual cells and molecules.

## Supporting Information

Figure S1 HPLC traces of LB control and *Pseudomonas aeruginosa* supernatant extracts.

Table S1 Quantification of the responses of *Pseudomonas aeruginosa, Escherichia col*i, and *Burkholderia dolosa* to different purified fractions.
